# An Exploratory Qualitative Study of Postgraduate Nursing Students’ Experiences of Using GenAI in Learning Nursing Leadership

**DOI:** 10.3390/nursrep16100352

**Published:** 2026-10-03

**Authors:** Jackie Hoi Man Chan, Khalil Yousef, Ibrahim Alananzeh, Sarah Hatahet

**Affiliations:** 1 Division of Health Sciences, Higher Colleges of Technology, Abu Dhabi P.O. Box 25026, United Arab Emirates; 2 School of Humanity, Social Science and Health, University of Wollongong in Dubai, Dubai P.O. Box 20183, United Arab Emirates; khalilyousef@uowdubai.ac.ae (K.Y.); ibrahimalananzeh@uowdubai.ac.ae (I.A.); sarahhatahet@uowdubai.ac.ae (S.H.); 3 School of Nursing, University of Jordan, Amman 11942, Jordan

**Keywords:** nursing leadership, GenAI, Bloom’s taxonomy, flipped learning, postgraduate education

## Abstract

**Background/Objectives**: Generative artificial intelligence (GenAI) presents immense potential to advance the flipped learning approach. Yet, it is under-explored. This study presents the postgraduate nursing students’ experience in using GenAI when learning nursing leadership, guided by Bloom’s taxonomy, in a flipped-learning approach. **Methods**: A descriptive qualitative design was employed, and individual semi-structured interviews were conducted with nine postgraduate nursing students who had completed a nursing leadership course delivered via a GenAI-integrated flipped-learning approach. **Results**: Three interconnected themes emerged: (1) GenAI supplements in-person learning, (2) Direct and Socratic questioning, and (3) Factors contributing to the GenAI learning experience. Students appreciated that GenAI helped them summarize and understand the learning material, thereby promoting meaningful in-class activities. Students highlighted direct and Socratic questioning as the major approaches in using GenAI and reported their perceived strengths and weaknesses. Notably, Socratic questioning—AI-driven thought-provoking dialog—was perceived to have helped develop critical thinking and promote deep learning. However, suboptimal prompting technique was the barrier, while channels for learning prompting and integrating it into the curriculum were facilitators. **Conclusions**: This study reveals an overall positive experience with the use of GenAI in flipped learning in postgraduate nursing education.

## 1. Introduction

Nursing leadership is a critical component of graduate nursing education. It prepares nurses to advance patient outcomes, guide healthcare teams, and lead organizational and system-level changes [1]. Nurturing nurse leaders extend far beyond the delivery of knowledge; it requires intentionally facilitating the development of self-directed learning and critical thinking skills [2]. These crucial skills will help nurse leaders adapt continuously and make data-driven decisions with empathy in an evolving healthcare environment increasingly sophisticated by advanced technology. Traditional teaching methods, such as didactic lectures, may provide a foundational understanding of knowledge on nursing leadership. However, it does not cater to diverse levels of expertise and students’ learning paces [3]. In addition, its passive nature positions students as recipients of information rather than active knowledge acquirers, limiting the potential for developing higher-order thinking (i.e., evaluating and creating according to Bloom’s cognitive learning taxonomy) [3]. This highlights the significance of an effective pedagogical approach that promotes self-directed learning and higher-order thinking in nursing leadership among postgraduate nurses.

Flipped learning provides an excellent environment for learning nursing leadership among postgraduate nursing students. A recent meta-analysis suggests that it may enhance self-directed learning and metacognitive thinking skills at one’s own pace among nursing students [2]. There are generally three phases in flipped learning: pre-class, in-class, and post-class. Students engage with asynchronous learning materials, including literature, videos, and web-based information, via an online learning management system (e.g., Moodle) before class. This self-paced learning provides the necessary background for more complex discussions and practical applications during in-class sessions, where learning is reinforced for higher-order cognitive skills. Subsequently, post-class activities and assessments, such as project presentations and analytical discussions, further consolidate understanding by offering students opportunities to reflect and apply [3]. Flipped learning empowers students to take ownership of their learning, allowing them to complete it at their own pace and on their own schedule. This approach aligns with the learning needs of postgraduate nursing students, as they are adults engaged in shift work. While flipped learning uses technology to support its successful implementation, GenAI presents immense potential to further enhance students’ learning experience.

GenAI is being widely adopted in nursing education. Emerging evidence suggests that GenAI-integrated flipped learning is effective in nursing education. A mixed-methods study demonstrated significant improvements in patient handover knowledge and oral performance among undergraduate nursing students when an AI chatbot was integrated into a flipped-learning environment [4]. However, several caveats should be carefully addressed when integrating GenAI into nursing education. First, studies revealed factual inaccuracies and failures in clinical judgment, highlighting the need for structured strategies to guide integration [5,6]. Second, nursing students from different countries may exhibit varying perceptions and related factors regarding the use of GenAI in learning. For example, the nursing students from the Arabic region demonstrated only a moderate level of perception towards the use of GenAI in nursing education, which was associated with their knowledge of GenAI and gender [7,8]. This may, in turn, affect students’ adoption of GenAI in learning. Third, complex prompting, unreadiness, and a lack of GenAI knowledge and exposure are reported as the most common difficulties encountered in healthcare students [9,10]. As such, gaining a deep understanding of nursing students’ experiences with GenAI is crucial to its successful implementation.

Despite the widespread use of GenAI in undergraduate nursing education, its use in postgraduate nursing education has been underreported and has been largely limited to research literacy and assignment preparation [11,12,13,14]. In addition, the evaluation of nursing students’ perceptions of GenAI in learning has largely relied on quantitative methods [15], yielding limited insight into what facilitates learning and how. This represents a gap in the qualitative understanding of postgraduate nursing students’ perceptions of GenAI in learning nursing leadership via a flipped learning approach. The aims of this study were to explore the experience of a GenAI-integrated flipped-learning approach in a nursing leadership course among postgraduate nursing students and to identify facilitators and barriers to integrating GenAI into postgraduate nursing education.

## 2. Materials and Methods

### 2.1. Design

This study employed a descriptive qualitative design to explore students’ in-depth experiences with GenAI in learning, focusing on their learning approaches and the barriers and facilitators they encountered. This study design was chosen because it is particularly well suited to investigating how individuals perceive and derive meaning from their experiences [16]. Individual semi-structured interviews were used to collect data and yield in-depth narratives [17]. The reporting followed the Consolidated Criteria for Reporting Qualitative Research (COREQ) [18].

### 2.2. Participants and Sampling

We utilized a convenience sample of graduate nursing students who enrolled in the Master of Nursing program at a private university in the United Arab Emirates. This study took place on an existing course, Leading Change in Healthcare, which was a core subject of the postgraduate nursing program. The inclusion criterion was achieving at least 70% course attendance to provide profound narratives of the learning experience via GenAI [17].

### 2.3. Development of a GenAI-Integrated Flipped Learning

The existing course, Leading Change in Healthcare, is currently delivered via a flipped learning approach. This course is a core subject of the Master of Nursing Program in a university in the UAE. The course had 4 modules and spanned 12 weeks. We employed multiple strategies to guide the integration of GenAI in this subject: (1) aligning university policy and guidelines on GenAI use; (2) ensuring pedagogical alignment; (3) promoting competency in GenAI prompting; and (4) conducting a pilot test [19]. The university is committed to embracing GenAI as a tool to enhance learning experiences and develop vital work-readiness skills. Concrete guidelines for teaching and learning, free access to Microsoft Copilot, and an abundance of online resources are available to empower nursing faculty and graduate nursing students in adopting GenAI in teaching and learning. Building on this GenAI policy, we enhanced the already employed flipped learning approach by integrating GenAI into teaching and learning in this nursing leadership subject.

Meanwhile, we encouraged the postgraduate nursing students to engage with the university-provided information on GenAI literacy, including basic knowledge and prompting techniques. This information was provided via various channels, including online seminars and websites. The principal investigator of this study was the lecturer who delivered the course and had extensive experience integrating educational technologies into nursing education. Therefore, the prompting techniques were also demonstrated during class time. Two specific prompting techniques were introduced: direct questioning and Socratic questioning. These two prompting techniques represent two learning modes: direct acceptance of knowledge (i.e., direct questioning) and building own knowledge through discussion with GenAI (i.e., Socratic questioning). Students may directly prompt Microsoft Copilot (Microsoft 365) for answers with recommended prompts. They were also encouraged to employ Socratic questioning via the guided learning mode in Microsoft Copilot (Microsoft 365), which, rather than giving direct answers, asks follow-up questions based on students’ responses. This Socratic questioning was particularly encouraged for understanding complex leadership concepts. The general prompt for this Socratic questioning would be “Employ Socratic questioning; do not give direct answers but guide me by asking questions based on my answers.” Although instructions for in-class activities and assessments were provided, students may choose their learning mode when using GenAI. Regarding GenAI tools, we encouraged students to use Microsoft Copilot (Microsoft 365), as it was provided by the University; however, ChatGPT-4o was also allowed for flexibility and easy access. The answers to the GenAI-enhanced learning activities were discussed and shared in class to address any misconceptions and possible hallucinations.

In addition, we employed the revised Bloom’s taxonomy [20] to guide the development of GenAI-enhanced learning activities for flipped learning. For example, the students were instructed to summarize different leadership styles as an in-class activity using Microsoft Copilot (Microsoft 365) (Understanding) and were also encouraged to generate an image using Microsoft Copilot (Microsoft 365) to help them reflect on their learning experience during class (Application). Table 1 presents the GenAI-integrated learning activities in different modules for the nursing leadership subject. For assessment, students were required to prompt Microsoft Copilot (Microsoft 365) for opinions on a healthcare-change statement and then critically analyze the GenAI-generated opinions (Evaluation). The assessments were designed to reflect students’ critical evaluation of the GenAI output. Table 2 presents the GenAI-integrated assessment.

### 2.4. Data Collection

The interviews were conducted in August 2025. Given the dual role of the first author as both course instructor and researcher, it was important to maintain researcher reflexivity, in which the researcher critically reflected on how their beliefs, experiences, assumptions, and position might influence recruitment and participants’ willingness to express negative views. In addition, there was a potential for inherent power imbalance and social desirability bias. To mitigate these issues, multiple strategies were adopted in data collection. First, eligible participants were invited to take part in semi-structured individual interviews upon course completion and the release of final grades. In addition, an independent research assistant who was not involved in teaching the subject conducted the interview via videoconference. Furthermore, the participants were assured that their participation was voluntary, that their identities would be kept anonymous, and that their academic evaluation would not be affected by their decision to participate or by the views they expressed during the interviews.

A semi-structured interview guide, which included open-ended questions, was used to facilitate the interview. Table 3 presents the interview guiding questions. The questions were presented to the participants, who were given a brief moment to reflect on them before the interview began. The participants were encouraged to express their feelings, experiences, and challenges freely. These predetermined questions provided a framework for interviews, and spontaneous follow-up questions were asked to probe for deeper insights. During the interview, non-verbal cues, such as facial expressions and pauses, were also recorded. Before the interviews concluded, the information collected was summarized and verified with the participants. Within 24 h of each interview, the audio recordings were transcribed and verified against the originals for accuracy.

Both researchers (JC and IA) agreed that no additional data were found at the 7th interview; therefore, 2 more interviews were conducted to confirm that data saturation had been achieved, indicating that no new thematic information emerged. The audio-recorded interviews were transcribed for analysis, and the field notes taken during and after the interviews were incorporated.

### 2.5. Data Analysis

The interview data were independently analyzed for thematic extraction by two researchers (JC and IA) using thematic analysis [21]. The process consisted of six steps: Familiarizing yourself with your data, generating initial codes, searching for themes, reviewing themes, defining and naming themes, and producing the report. In the first step, the researcher immersed in the data by reading repeatedly at least three times of all the transcripts to build familiarity with the depth and breadth of the content, thereby searching the meaning and pattern in the data. We identified three major patterns in the data: how GenAI contributed to learning, the approaches students adopted when using GenAI, and the factors contributing to their experience in using GenAI. Then, the researchers generated codes for meaningful and interesting features across the data set. “Modify the generated output” and “Explain leadership concepts” were examples of some of the codes generated. Next, relevant codes were grouped together to form potential themes, and different levels of code within a theme were considered to form subthemes. For example, “Modify the generated output” and “Explain leadership concepts” were grouped together under the major theme “GenAI supplements in-person learning” because both demonstrated how GenAI contributed to learning. However, these two codes represented two ends, leading to the emergence of two subthemes which were “Facilitating self-learning prior to the in-person workshop” and “GenAI–human collaboration”. After that, the coded data extracts within a theme were reviewed to ensure coherent pattern and accuracy. Defining and naming of each theme was done to clarify the meaning and scope of each theme. The last step involved writing up the story the data told within and across themes in a concise, coherent, logical, and non-repetitive manner. All the data were managed in the software Microsoft Excel (version 2021).

### 2.6. Rigor of the Study

To ensure the trustworthiness of the study, several strategies were used, including credibility, dependability, transferability, and confirmability [22]. First, credibility was established through iterative engagement with the data, collaborative data analysis among researchers, and the use of representative quotations. Dependability was assured through a transparent and systematic analytic process, including coding (classification of content), condensation (summary of data), and abstraction (grouping data under subheadings). While transferability was addressed through clearly defined selection criteria and comprehensive demographic descriptions, confirmability was further supported through collaborative discussions and ongoing reflexivity within the research team. Strategies were employed to maintain researcher reflexivity during data analysis. Reflexive field notes were taken to record the researcher’s thoughts to help identify how personal perspectives may influence the research. In addition, member checking was maintained throughout the research process to minimize double hermeneutics, whereby participants interpret their own learning experiences, and the researcher subsequently interprets these accounts.

### 2.7. Ethical Considerations

Ethical approval (ref. no. 2025/193) was obtained from the university’s research ethics committee before the study started. The participants were approached and invited to participate upon completion of the course and release of the course’s final grades. Written information about the study was given and verbally explained. Voluntary participation and the right to withdraw without academic consequences were highlighted. Ample time (minimum 24 h) was provided to consider before consenting to the study.

## 3. Results

A total of 12 postgraduate nursing students enrolled in the course, and nine students consented to participate in the semi-structured interview individually. Those who did not consent to the study reported busy work and had no interest in participating. All participants were de-identified in accordance with confidentiality principles, and pseudonyms (R1–R9) were used in place of real names. All participants were female and aged 24 to 37 years. They were all registered nurses with 3-15 years of clinical experience. Their clinical expertise varied, including medical, surgical, obstetrics, gynecology, psychiatry, and accident and emergencies. Their experience with GenAI ranged from 5 to 24 months, and ChatGPT-4o was the only GenAI tool used outside class time. Their reason for using GenAI included personal reasons, learning, and entertainment. Table 4 presents the characteristics of the participants. The total duration of all the interviews was 193 min, averaging about 24 min per interview.

Following the analysis of the interviews, 122 codes were identified and subsequently categorized into three themes with six subthemes. The three themes that described the learning experience of nursing leadership among the graduate nursing students were (1) GenAI supplements in-person learning, (2) Learning approaches via GenAI, and (3) Factors contributing to the GenAI learning experience. The analysis and themes were sent back to the participants for comments, and they agreed with the findings. Table 5 presents the themes, subthemes, and the number of codes identified.

### 3.1. Theme 1: GenAI Supplements In-Person Learning

All the students highlighted the supplementary role of GenAI in learning, indicating that it could support, but not replace, human learning. Although GenAI provided quick answers and helped in understanding concepts, it was unable to provide real-life experiences from different clinical settings. Students appreciated the teachers’ explanation and the experience shared during the in-class session. They also enjoyed peer sharing, realizing that the leadership knowledge and principles could be generalized to different nursing specialties and hospital settings. To them, GenAI was perceived as having helped them prepare cognitively, enabling them to participate meaningfully in the class. Two subthemes emerged, further elaborating on how GenAI supplements human learning.

#### 3.1.1. Facilitating Self-Learning Prior to the In-Person Workshop

Prior to attending in-person workshops, all students used GenAI to promote self-learning of learning materials. Therefore, they could well prepare themselves for meaningful discussion and experience sharing among students and teachers during in-person workshops. All the students employed GenAI to facilitate self-learning, including checking for definitions, summarizing the literature and connecting concepts.

*‘I prefer to prepare myself before the workshop because I will understand the concept…I use ChatGPT outside the class, to support my understanding…in the class there will be discussion with my colleague, the teacher will bring personal experience…real-life examples…and the human interaction. *

(R2)

*‘I use ChatGPT for quick definitions about the types of leadership, attitude of inquiry and give me extra help to identify other articles. Sometimes I have two concepts, and I don’t know how they connect. I asked ChatGPT to analyse how concept A is connected to concept B.’ *

(R1)

*‘We have covered a lot of leadership styles, and using GenAI, it helped me have a clearer vision of leadership. And some of the leadership styles need practical examples, so GenAI has helped me a lot in understanding the components and how it can be applied practically in our nursing practice.’ *

(R7)

#### 3.1.2. GenAI–Human Collaboration

Beyond preparation, all the students employed GenAI to supplement their learning through scaffolding, enabling them to create new content based on what they had learned. For example, while contemplating a change proposal, one of the assessments, students identified gaps in clinical practice at the work facility. They then employed GenAI to suggest change models and build the generic change structure. Subsequently, they modified the GenAI output by adding details aligned with their facility’s context. They appreciated this GenAI–human collaboration.

*‘I didn’t ask GenAI to help that time because I had an idea from work, from my colleague’s collaboration. It’s from my observation and my work environment; I noticed that there’s a gap. Then, I check what the answer [model] is from ChatGPT, and try to modify it, or to fit with the context from our facilities and to the idea in the proposal which we want to use. So at least it’s helped us to get the best model, or framework. So we can use it in the implementation’ *

(R5)

### 3.2. Theme 2: Learning Approaches via GenAI

Although instructions for in-class activities and assessments were provided, students may opt for different learning modes when using GenAI. Students reported two primary approaches to learning with GenAI: direct questioning and Socratic questioning. They perceived strengths and weaknesses for each approach.

#### 3.2.1. Direct Questioning

All students attempted to obtain answers and information directly from GenAI, which generated specific responses to their questions. They reported that this approach helped them understand and analyze complex concepts quickly. All students recalled that the benefits of this approach included user-friendliness, enhanced efficiency, and the ability to manage a large volume of reading materials while maintaining full-time employment and life commitments. However, six students reported the downsides, including overreliance and adopting the output without criticizing the content. Additionally, half of the students reported inaccuracies and irrelevance in the output as emotional and contextual factors were not accounted for.

*‘I will ask ChatGPT, okay, what does this kind of leadership mean? Um, or what do we do with it? Give me examples. And then he [ChatGPT] will provide me with all such information. I use it to make direct answer, so I can understand the concept quickly. However, there are time that answers are incoherent’ *

(R3)

*‘I would just copy-paste the…. the title, the authors, and then I would just say, summarize so I can get the whole idea in short period of time…Uh… just to save time when I’m really struggling with schedule [work schedule], family, and all.’ *

(R8)

*‘We become much more efficient because the output of ChatGPT is very quick…to have a concise, you know output. Instead of reading sometimes 10 or 20 pages. Biggest concern is that it can reduce our critical thinking and independent learning…because I adapt the answer on ChatGPT. Yeah, sometimes I use it just to get the answers and copy them directly.’ *

(R1)

*‘It’s not 100% accurate. I had to check the applicability in my area. It does not fully capture the ethical and cultural dimensions of leaders. The real setting…daily life experiences…it’s about emotions. It’s very linked to emotions. ChatGPT cannot always give you the emotional understanding and those reactions that is happening.’ *

(R7)

#### 3.2.2. Socratic Questioning (Simulated Discussion with GenAI)

Half of the students reported using Socratic questioning to complete the class activities and assessments. Rather than asking GenAI to provide answers directly, half of the students prompted it to ask a series of reflective questions corresponding to their answers. They appreciated that this approach helped them identify what they know and what they don’t. They also highlighted that the subsequent questions GenAI asked, based on their responses, guided them to build the answers themselves. Half of the students recalled that this approach helped them explore the topic more deeply. They also felt that this approach helped develop critical thinking skills. However, two students highlighted that this learning approach may need considerable adaptation and is time-consuming.

*‘I told [prompted] GenAI: don’t give me the answer; guide my learning. For [learning] leadership, GenAI will give me questions about that topic: What do you know about the definition? What do you know about examples? Then I am writing [answering]. Then, GenAI will ask me more questions [corresponding to my answers], for example, something I don’t know. Sometimes I don’t know the answer, then GenAI will give me some hints. I was also thinking this one for [analysing] the supporting and opposing data. For example, psychological factor will be important than the other one. However, it takes more time to use and adapt to this learning style’ *

(R6)

*‘I feel like we are collaborating, like you are having discussion with somebody. I feel it is developing my critical thinking because when I was using GenAI to guide my learning… I have in-depth understanding of the content…I am discovering other points that I didn’t think about it and that is very nice.’ *

(R7)

### 3.3. Theme 3: Factors Contributing to GenAI Learning Experience

Several barriers and facilitators regarding a successful GenAI learning experience emerged from the narratives. All students highlighted that the preparation for adopting GenAI, including the knowledge and skills, was paramount. In addition, they appreciated the GenAI-integrated in-class activities, and the clear instructions for the GenAI-integrated assignment are integral to the unique learning experience it enables.

#### 3.3.1. Complex Prompting and Suboptimal Prompting Technique

The complexity of prompting and students’ suboptimal prompting techniques appeared to be barriers to students’ learning when using GenAI. GenAI requires specific instructions, known as prompts, to produce the output students intend. However, students reported suboptimal prompting techniques during the learning process. Students consistently reported these barriers,

*‘There is prompting technique, and there is a way to ask for the information from the GenAI. There are some criteria writing it, so you can get exactly what information you want. And it was difficult, because that time we don’t know about the prompt technique and we try multiple times till we get it.’ *

(R5)

*‘ChatGPT does not always understand the context so I need to give the GenAI the right prompt…to make it clearer for ChatGPT about the context that I’m working at so it can give more realistic solutions. I have multiple attempts trying to get the right solution, and proposal. If you have given a wrong prompt, this will lead to a wrong response.’ *

(R7)

#### 3.3.2. Prompt Learning and Integration into Curriculum

Providing various channels for prompt learning appeared to facilitate learning with GenAI. Reported by the three students, in-class instruction, web-based information, and online seminars were the primary channels for prompt learning. Additionally, integrating GenAI into the curriculum emerged as another facilitator. Two students highlighted that they learnt prompting while practicing and applying it during in-class activities and completing assignments. They also felt empowered to use GenAI to complete assignments when clear rubrics were provided.

*‘This semester the teacher introduced to us and from the one in the link from the LinkedIn and from the university website. The lecture [online seminar] from the university…Actually, I attended, and they showed us different ways of using the GenAI. But before that, really, we were not knowing that there is some way to write the prompt. We’re just simply asking ChatGPT.’ *

(R5)

*‘The one when we did… the communication table, and she [the teacher] showed us to use the GenAI to create that during the … [workshop].’ *

(R5)

*‘Actually, it was incorporated in our rubrics…it was incorporated in our assessment… how to apply the right prompt [to complete assignment].’ *

(R7)

## 4. Discussion

This study explored the postgraduate nursing students’ experiences in using GenAI when learning nursing leadership. The findings provided an in-depth understanding of how this emerging technology is being perceived in postgraduate nursing education. All study participants were adult learners with full-time employment. They represent learners with diverse backgrounds, expertise levels, and learning curves, and therefore specific learning needs. A student-centered approach becomes crucial to facilitate their learning journey. The findings revealed postgraduate nurses’ experiences with GenAI-integrated flipped learning in nursing leadership. It also revealed the strengths and weaknesses of the learning approaches with GenAI. The students appreciated the efficiency and personalized guidance from GenAI, which could meet their unique learning needs. This is aligned with the findings from a recent cross-sectional study about GenAI in postgraduate nursing programs [15] and higher education in general [23].

Meanwhile, our findings are partly aligned with current criticisms of GenAI in undergraduate nursing education, including overreliance and limited critical thinking. These downsides were highlighted when students used direct questioning to prompt output from GenAI. Recent reviews suggested that the rapid responses provided by GenAI encouraged direct acceptance without critical thinking, leading to overreliance and potentially metacognitive laziness [23,24,25]. Additionally, direct questioning is prone to bias and errors when learners do not critically evaluate the output generated. Nursing educators and researchers should therefore be cautious when integrating GenAI into teaching and learning and avoid this pitfall.

Our findings also provided insights into another approach to using GenAI: Socratic questioning, which was perceived as facilitating critical thinking and deeper learning. Originating with Greek philosophers, Socratic questioning is an ancient approach to learning that relies on debate and discussion between students and teachers. The teachers ask a series of thought-provoking questions to clarify and delve deeper, based on the students’ responses. Such inquiry-based dialog prompts students to construct answers through logical reasoning, thereby developing critical thinking [26]. Facilitated by GenAI, Socratic questioning was used to create a simulated dialog with students and guide learning in the current study. Emerging evidence demonstrates the effectiveness of Socratic questioning in nursing education, including simulation debriefing [27], development of critical thinking [28,29], and moral reasoning [30]. Although the findings from the current study did not provide evidence of the effectiveness of Socratic questioning, students’ perceptions were reported, contributing to the understanding of the use of this learning approach with GenAI.

In the context of nursing leadership, decision-making and strategic planning extend beyond mere acceptance of knowledge. Critical thinking and empathy are paramount in making human-centered decisions and navigating the nursing profession in the world of advanced technology. Therefore, continuous questioning, awareness, and reflection are crucial for graduate nursing students to develop effective leadership skills. Building on students’ work experience and expertise, Socratic questioning may be employed to provide a personalized pathway that promotes deeper understanding and reflection in the context. Literature suggests that successful delivery of GenAI-enhanced Socratic questioning hinges on students’ initiation [31]. Graduate students may greatly benefit from this approach as they are adult learners who are self-motivated and willing to take charge of their own learning journey. Meanwhile, the current findings revealed considerable adaptations and time constraints as challenges of using Socratic questioning. These may explain the frustration and student disengagement resulting from ineffective dialog, according to a previous study [32]. Amid the inconsistencies, further interventional studies, with rigorous designs such as randomized controlled trial, are warranted to provide empirical evidence on its effectiveness before drawing any conclusion. Therefore, facilitating Socratic questioning via GenAI in learning may pose a challenge for nursing educators and researchers.

Currently, there are GenAI tools that can integrate Socratic questioning into learning, including Gemini Notebook (version 1.51.1), ChatGPT (GPT-5.5), and Google Gemini’s (version 2.5 pro) guided learning mode. These GenAI tools are user-friendly and can be prompted to use Socratic questioning and to provide reflective questions based on learners’ understanding. In particular, the learning management system, Blackboard Ultra, provides an AI conversation mode that provides Socratic questioning “https://www.blackboard.com/blackboard-lms-ai (accessed on 4 April 2026)”. Therefore, faculty may create interactive, AI-driven, and formative learning experiences within courses. However, empirical evidence in evaluating its effectiveness is lacking. How to balance the use of direct and Socratic questioning with GenAI to achieve a synergistic effect remains to be explored.

It is worth noting that we have implemented several strategies in designing the integration of GenAI into the current course. They included aligning with university policy and guidelines on GenAI, ensuring pedagogical alignment, and promoting prompting competency [19]. The learning experience demonstrated in this study reinforced the importance of careful, strategic planning during implementation. It is therefore highly recommended to adopt these strategies for integrating GenAI into nursing education in the future.

In a recently published Editorial [31], the authors suggest that there is a scarcity of evidence on the use of Socratic questioning in nursing education. To our knowledge, this study is perhaps one of the few that provides information on the potential of GenAI-based Socratic questioning among postgraduate nursing students. This research adds to the growing body of knowledge around GenAI-integrated flipped learning in nursing education. It also provides nurse educators and researchers with insights into how GenAI may be pedagogically aligned to support learning. The findings provide insights for nursing faculty and researchers on the use of GenAI-integrated flipped learning in nursing leadership among postgraduate nursing students. Notably, AI-driven Socratic questioning has the potential to promote critical thinking in the era of AI. Further rigorous, large-scale interventional studies are necessary to evaluate the effectiveness of Socratic questioning via GenAI on students’ critical thinking, thereby providing the best evidence to guide the adoption of GenAI in nursing education.

### Limitations

Nevertheless, this study has limitations, and the reader should interpret the findings with caution. First, the participants were selected from a pool of 12 students enrolled in a single course at a single institution, which may limit the generalizability of the findings to broader international nursing cohorts. Second, all participants were female, lacking gender diversity of perspective. Third, because all students used only ChatGPT-4o and Microsoft Copilot (Microsoft 365), the findings may not fully represent experiences with other GenAI tools. Lastly, the findings only reflect the perception and experiences of the nursing students in learning with GenAI. The findings did not demonstrate any quantitative evaluation on the use of GenAI.

## 5. Conclusions

This study explored the experiences of postgraduate nursing students in a GenAI-flipped learning approach to learning nursing leadership. The findings suggested an overall positive experience in using GenAI to supplement in-person learning by facilitating students to understand, evaluate, analyze and create during the learning journey. Students reported using direct questioning and Socratic questioning as approaches when learning with GenAI. Perceived user-friendliness and enhanced efficiency were the reported strengths of direct questioning, while overreliance, inaccuracies, and direct acceptance with minimal criticism were its perceived downsides. Promoting deeper learning and critical thinking skills were the perceived benefits of Socratic questioning, while considerable adaptations and time constraints were its reported downsides. Complex prompting was revealed as a barrier, while intended curriculum integration and various channels for learning prompting were the facilitators of GenAI-integrated flipped learning. Further interventional studies with rigorous designs are paramount for providing empirical evidence for this novel pedagogical approach.

## Figures and Tables

**Table 1:** GenAI integrated in-class activities across different modules.

Modules	Topics	Learning Objectives	GenAI-Integrated In-Class Activities	Bloom’s Taxonomy Level to Achieve
1	Leadership styles and behaviors	Critically appraise various leadership styles and behaviors and relate them to individual or organizational change in the healthcare environment	Explain ideas or concepts Provide a summary of a leadership model using the provided literature and illustrate with your own clinical experience. Recommended prompt: Generate a summary of [a leadership style] based on the literature provided. Connect concepts with experience Generate a picture that illustrates a previous leader who impressed you in the clinical setting and explains the leadership behaviors. Recommended prompt: Generate a picture to illustrate a previous nursing leader according to the description: [an experience with the previous nursing leader].	Understand Apply
2	Change models	Critically appraise different change models and factors for change in the healthcare environment	Explain ideas or concepts Generate a summary of a change model using the provided literature and illustrate it with your own clinical experience. Possible prompt: Generate a summary of [a change model] based on the provided literature. Justify the arguments Critique the arguments suggested by GenAI and provide justifications. Recommended prompt: Analyze the uploaded case scenario and suggest the benefits and downsides of the change strategies used.	Understand Evaluate
3	Planning and communicating change	Propose transformational leadership skills necessary to implement organizational change and act as a change agent Formulate and defend a plan to lead an innovative change within the healthcare environment	Justify a change model for an identified change Critique a change model suggested by GenAI for an identified change in a clinical setting. Recommended prompt: Suggest a change model based on an identified change described here: [description of an identified change in a clinical setting]. Justify communication strategies Create and critique the communication content suggested by GenAI for the identified change mentioned above. Recommended prompt: Structure a table to map communication contents across different levels of change (i.e., Awareness, Engagement, Acceptance, Action), and levels of involvement (i.e., Tell, Sell, Consult, Join). The communication content should be based on an identified change described here: [description of an identified change in clinical setting].	Evaluate Create
4	Implementing and evaluating change	Formulate and defend a plan to evaluate an innovative change within the healthcare environment	Justify an evaluation model for the structured planning of an identified change Critique an evaluation model suggested by GenAI for a structured plan of an identified change built in Module 3. Recommended prompt: Structure an evaluation plan based on the uploaded file, which presents a structured plan of an identified change in a clinical setting.	Evaluate

Note. Words in [] is subject to the decision of the students.

**Table 2:** GenAI integrated assessment.

Assessment	Guidelines	Bloom’s Taxonomy Level to Achieve
Case analysis	**Part I**—Use a GenAI tool to generate a response that agrees and disagrees with the provided statement. *‘Research suggests that change has both situational and psychological aspects, and ignoring either will result in a non-sustainable situation, wherein the organization is in a constant cycle of change implementation without achieving desired results.’* **Part II**—Identify peer-reviewed publications that support the GenAI responses generated above, and discuss with examples.	Evaluate
Presentation of change proposal	**Part I**—Use GenAI tool to generate the structure and components of the proposed change identified from the clinical setting. **Part II**—Based on the structure of the change proposal generated by GenAI, critically examine the GenAI output and customize it to reflect the needs of the students’ organization and the proposed change.	Create

**Table 3:** Semi-structured interview guiding questions.

What is your experience of learning with use of GenAI in this subject?
2. What are the best features of GenAI in learning in this subject?
3. What are the worst features of GenAI in learning in this subject?
4. What has helped you to engage or inhibited you from engaging in the use of GenAI in learning in this subject?
5. What are your suggestions to improve the use of GenAI in this subject?

**Table 4:** Interview characteristics of participants (n = 9).

Code	Gender	Age (Years)	Profession	Clinical Experience (Years)	Clinical Specialty	Experience of Using GenAI (Months)	GenAI Tools Used	Reason for GenAI Use
R1	Female	24	RN	3	OT	12	ChatGPT-4o, Microsoft Copilot *	Personal reason, learning
R2	Female	25	RN	3	Gynecology	5	ChatGPT-4o, Microsoft Copilot *	Learning, entertainment
R3	Female	37	RN	8	Surgical	6	ChatGPT-4o, Microsoft Copilot *	Personal reason, learning
R4	Female	24	RN	3	Psychiatry	12	ChatGPT-4o, Microsoft Copilot *	Personal reason, learning, entertainment
R5	Female	37	RN	15	Surgical	13	ChatGPT-4o, Microsoft Copilot *	Learning
R6	Female	26	RN	4	ED	24	ChatGPT-4o, Microsoft Copilot *	Personal reason, learning
R7	Female	28	RN	5	Medical	19	ChatGPT-4o, Microsoft Copilot *	Learning
R8	Female	25	RN	3	Obstetric	NA	ChatGPT-4o, Microsoft Copilot *	Learning, entertainment
R9	Female	26	RN	5	Surgical	5	ChatGPT-4o, Microsoft Copilot *	Learning, entertainment

Note. ED: Emergency department, NA: not available, OT: operation theater, RN: registered nurse, *: all Microsoft Copilot was Microsoft 365.

**Table 5:** Total number of codes in each theme.

Theme	Subtheme
GenAI supplements in-person learning	Facilitating self-learning prior to the in-person workshop
	GenAI–human collaboration
Learning approaches via GenAI	Direct questioning
	Socratic questioning
Factors contributing to GenAI learning experience	Complex prompting and suboptimal prompting technique
	Prompt learning and integration into curriculum

## Data Availability

Data can be obtained from the corresponding author.

## Public Involvement Statement

No public involvement in any aspect of this research.

## Guidelines and Standards Statement

This manuscript was drafted against the Consolidated Criteria for Reporting Qualitative Research (COREQ).

## Abbreviations

The following abbreviations are used in this manuscript:

GenAI	Generative artificial intelligence
